# Supplementary material for: MetaRibo-Seq measures translation in microbiomes
Source: Nat Commun. 2020 Jun 29;11:3268. doi: 10.1038/s41467-020-17081-z (PMC7324362; doi:10.1038/s41467-020-17081-z)
Supplement: Supplementary file 10 — Supplementary Data 7 [file 41467_2020_17081_MOESM10_ESM.zip › File2/Confidence_High_Taxonomy/125538_out.krona.html]

Javascript must be enabled to view this page.

members
magnitude
magnitudeUnassigned
count
unassigned
taxon
rank

125538\_out

3

superkingdom
2
3

1239
3
phylum

class
3
186801

order
186802
3

186806
2
family

genus
2
1730


SRS044535\_contig\_number\_16344
species
1
39485

142586
1
species

SRS055966\_contig\_number\_contig-100\_8806.8807

1
186803
family

genus

SRS1055049\_contig\_number\_contig-100\_27983.27984
841
1
